# Iloprost Attenuates Oxidative Stress-Dependent Activation of Collagen Synthesis Induced by Sera from Scleroderma Patients in Human Pulmonary Microvascular Endothelial Cells

**DOI:** 10.3390/molecules26164729

**Published:** 2021-08-05

**Authors:** Roberta Giordo, Duong Thi Bich Thuan, Anna Maria Posadino, Annalisa Cossu, Angelo Zinellu, Gian Luca Erre, Gianfranco Pintus

**Affiliations:** 1 College of Medicine, Mohammed Bin Rashid University of Medicine and Health Sciences, Dubai 505055, United Arab Emirates; robertagiordo2000@yahoo.it; 2 Faculty of Biochemistry, College of Health Sciences, VinUniversity, Vinhomes Ocean Park, Gia Lam District, Hanoi 132002, Vietnam; duongthuan@huemed-univ.edu.vn; 3 Department of Biomedical Sciences, University of Sassari, 07100 Sassari, Italy; posadino@uniss.it (A.M.P.); cossuannalisa@libero.it (A.C.); azinellu@uniss.it (A.Z.); 4 Rheumatology Unit, Department of Clinical and Experimental Medicine, University Hospital (AOUSS) and University of Sassari, 07100 Sassari, Italy; 5 Department of Medical Laboratory Sciences, College of Health Sciences and Sharjah Institute for Medical Research, University of Sharjah, University City Rd, Sharjah 27272, United Arab Emirates

**Keywords:** systemic sclerosis, oxidative stress, collagen synthesis, iloprost

## Abstract

Endothelial cell injury is an early event in systemic sclerosis (SSc) pathogenesis and several studies indicate oxidative stress as the trigger of SSc-associated vasculopathy. Here, we show that circulating factors present in sera of SSc patients increased reactive oxygen species (ROS) production and collagen synthesis in human pulmonary microvascular endothelial cells (HPMECs). In addition, the possibility that iloprost, a drug commonly used in SSc therapy, might modulate the above-mentioned biological phenomena has been also investigated. In this regard, as compared to sera of SSc patients, sera of iloprost-treated SSc patients failed to increased ROS levels and collagen synthesis in HPMEC, suggesting a potential antioxidant mechanism of this drug.

## 1. Introduction

Systemic sclerosis (SSc), also called scleroderma, is a chronic multisystemic and autoimmune disorder characterized by vasculopathy and progressive fibrosis of the skin and visceral organs, such as lungs, heart, and digestive tract [1,2]. In this regard, predisposing factors such as environmental and genetic may affect the susceptibility to scleroderma. These factors affect innate and adaptive immunity functions ultimately promoting inflammation and endothelial dysfunction, two phenomena that precede the SSc-associated fibrotic process [3,4,5,6]. Despite the enormous scientific effort in studying SSc etiology and pathogenesis, the overall etiopathogenic mechanisms picture remains obscure [7]. Along with the essential contribution of the genetic background and immune system [3,4,5,6], the last two decades’ studies have highlighted an important role of oxidative stress in SSc development. SSc patients present an altered balance between oxidant and antioxidant states, which is defined as oxidative stress [8]. Indeed, increased oxidative biomarkers and decreased anti-oxidative ones have been reported in SSc patients, clearly indicating a linkage between an altered redox homeostasis and this disease [8,9,10,11]. Indeed, as compared to healthy donor cells, several cell types coming from SSc patients, such as fibroblasts, monocytes, T lymphocytes, and erythrocytes, showed higher levels of reactive oxygen species (ROS) [12,13,14,15,16]. Moreover, fibroblast, endothelial cells, and vascular smooth muscle cells exposed to sera of SSc patients have a higher level of ROS as compared to healthy subject sera exposition [9,17]. In this regard, it now becoming evident that an overproduction of ROS can activate all cellular SSc targets (endothelial cells, fibroblasts, immune cells, and vascular smooth muscle cells) and trigger the inflammatory, autoimmune, and fibrotic processes defining SSc pathology [8,18]. Although the molecular mechanisms are unclear, vascular dysfunction appears to be an early SSc event, as most of the patients develop Raynaud’s phenomenon (RP), a small vessel disorder preceding onset disease progression, which involves both non-oxidative and oxidative pathways [19]. In this context, while non-oxidative pathways are mostly associated with vascular tone control, the oxidative ones prompt increased ROS production, triggering the excessive deposition of collagen and extracellular matrix (ECM) components leading to tissue fibrosis [8,19,20]. Indeed, during these early disease events, different ROS generating immune mediators, such as transforming growth factor beta (TGF-β), connective tissue growth factor (CTGF) endothelin (ET-1), interferons, and cytokines, are released and contribute to the disease onset and progression [8,19,20,21].

Intravenous iloprost, a stable analog of prostacyclin (PGI2), is a first-line therapeutic alternative for treating both SSc first manifestation and disease progression [22,23]. Iloprost’s beneficial effects (vasodilation, anti-platelet aggregation, cytoprotection, and immunomodulation) are largely due to the modulation of small vessel vasculopathy, one of the key factors in SSc disease [23,24]. In addition, many studies have reported the antioxidant effect of iloprost infusion in SSc patients, but the molecular mechanisms underlying it remain unknown [25,26]. We hypothesized that pro-oxidant circulating factors may trigger SSc-fibrotic process early events, such as oxidative stress and collagen I synthesis, and that the antioxidant properties of iloprost may counteract this course, thus ameliorating the SSc patients’ conditions. To verify our research question, we investigated ROS production and collagen I synthesis in human pulmonary microvascular endothelial cells (HPMECs) exposed to the sera of healthy subjects, SSc patients, and iloprost-treated SSc patients.

## 2. Results and Discussion

**Figure 1:**
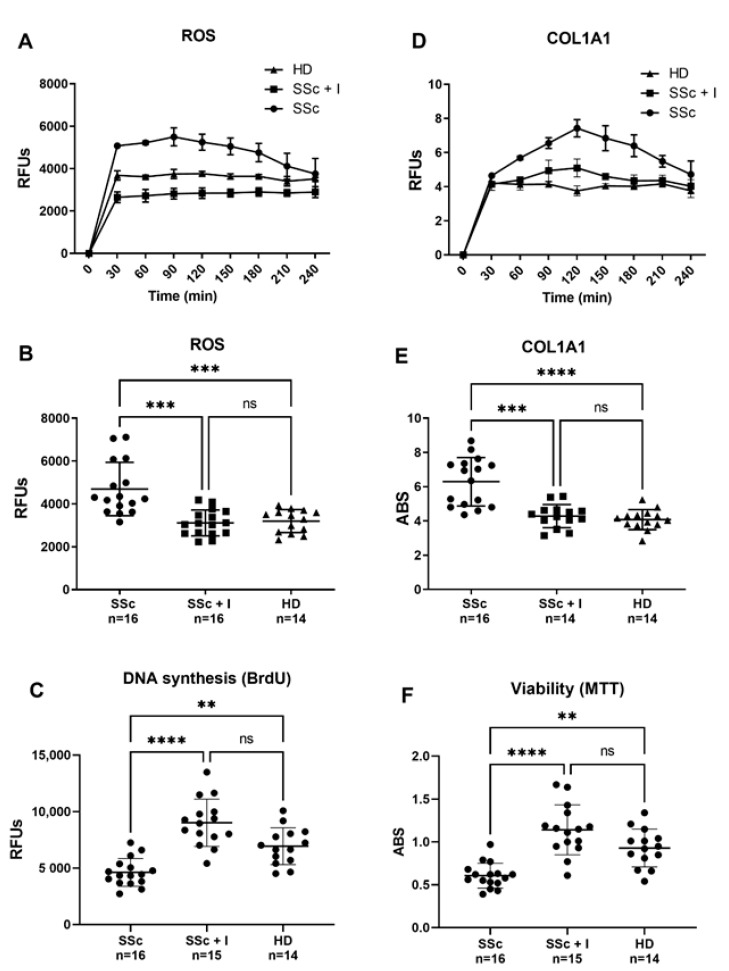
(**A**,**B**) Effect of SSc and SSc iloprost-treated sera on human pulmonary microvascular endothelial cells (HPMECs) intracellular ROS levels. Before sera treatment, subconfluent HPMECs were loaded with 10 μM of H_2_-DCFDA, and then cultured in basal medium containing 10% (*v*/*v*) of sera from SSc patients (SSc), sera from iloprost-treated SSc patients (SSc + I), and sera from healthy donors (HD). Variations in intracellular ROS levels were kinetically determined in a 4 h time-course (**A**) and values at the first hour were used for comparison (**B**). Fluorescence data were normalized for protein content and expressed as relative fluorescence units (RFUs). (**D**,**E**) Effects of SSc and SSc iloprost-treated sera on HPMECs collagen promoter activity. Before sera treatment, subconfluent HPMECs were transduced with lentiviral particles, obtained from the pCOL1A1-LV-tGFP and EFα-LV-FP602 lentivectors, and then cultured in basal medium containing 10% (*v*/*v*) of sera from SSc patients (SSc), sera from iloprost-treated SSc patients (SSc + I), and sera from healthy donors (HD). Variations of COL1A promoter activation were kinetically followed for 4 h (**C**) and values at 1 h (steady state) used for comparison (**D**). Data are normalized for transduction efficiency by reporting the ratio of pCOL1A1-LV-tGFP to EFα-LV-FP602 relative fluorescence units (RFUs) (green/red fluorescence). (**C**–**F**) Effects of SSc and SSc iloprost-treated sera on HPMECs DNA syntheses and viability. Subconfluent HPMECs were cultured for 24 h in basal medium containing 10% (*v*/*v*) of sera from SSc patients (SSc), sera from iloprost-treated SSc patients (SSc + I), and sera from healthy donors (HD). Cells were then assessed for DNA synthesis (**C**) and cell viability (**F**), as described in materials and methods. Horizontal lines indicate the mean value and standard deviation range. Kruskall–Wallis one-way analysis of variance, followed by a post-hoc Dunn’s test for multiple comparisons, was used to detect differences among studied groups in Figure 1B,D. All statistical analyses were performed using GraphPad Prism version 9.00 for Windows (GraphPad Software, San Diego, CA, USA), and *p*-values < 0.05 were considered to be statistically significant. **, between *p* = 0.0043 and 0.0082; ***, between *p* = 0.0001 and 0.0009; ****, *p* ≤ 0.0001; ns, no significant differences have been detected.

Reactive oxygen species (ROS) production by skin, visceral fibroblasts, and endothelial cells (ECs) has been suggested as an SSc background [27,28]. Indeed, SSc-associated oxidative stress causes both activation and endothelium damage, ultimately leading to vascular complications [28]. As ROS can trigger cell activation, and since ECs are one of the primary targets in SSc, endothelial dysfunction is likely to be the initiator of the cascade of events leading to SSc typical vascular remodeling [19,20]. ECs can, thus, stimulate important mediators, such as growth factors, cytokines and inflammatory agents, which play a pivotal role in the onset and progression of fibrosis [29]. Another SSc hallmark is the excessive deposition of ECM components such as collagen, caused by either increased synthesis or alterations in the degradation mechanisms [30]. In this regard, besides fibroblast and smooth muscle cells, ECs are also an important source of collagen synthesis, although less mentioned [20,31]. In comparison to sera from HD, here, we demonstrate that sera from SSc patients induce both abnormal ROS production and increased collagen synthesis in HPMECs, confirming the hypothesis that pro-oxidant circulating factors may trigger SSc early fibrotic events in an ROS-dependent fashion. Intracellular ROS levels and COL1A1 promoter activity has been kinetically determined in a 4 h time-course (Figure 1A–D), and values at the first hour have been used for comparison (Figure 1B–E). As depicted in Figure 1B, sera from SSc patients significantly increased intracellular ROS levels, compared with HD sera. In the same way, SSs sera-induced activation of collagen synthesis, which prominently occurs in the first hour, resulted significantly higher than that induced by HD sera (Figure 1E). Iloprost’s vasculoprotective properties have been also reported in a recent paper showing its ability to preserve vascular function by stabilizing endothelial adherens junctions, increasing tubulogenesis and barrier function, ultimately reducing ECs shift toward collagen-producing/αSMA-positive cells [32]. In agreement with these data, our current findings report the ability of Iloprost to counteract both ECs damage (Figure 1C–F) and collagen synthesis (Figure 1E) elicited by sera of SSc patients.

Antioxidant-based therapies represent valid support in the treatment of SSc-associated vascular diseases. In this contest, iloprost infusion appears as the most suitable therapy for treating RP and SSc-related vasculopathy [23,33]. Several evidences suggest iloprost’s antioxidant effect at the basis of its clinical benefits [26], but the molecular mechanisms underlying this antioxidant activity are nearly unknown. Our data indicated that sera of SSc patients increase ROS levels in HPMECs. In contrast, sera of Iloprost-treated SSc patients elicits intracellular ROS levels similar to those produced by HD sera (Figure 1B). Similarly, an SSc-induced increase in COL1A1 synthesis resulted in significantly attenuated HPMECs exposed to sera of iloprost-treated SSc patients, suggesting collagen synthesis activation as a potential target of the loprost therapeutic effect (Figure 1D). A possible explanation of the iloprost treatment’s beneficial effect might be the ability to decrease the levels of circulating pro-oxidant and pro-fibrotic factors, which has been reported as increased in the SSc sera [28,34,35,36,37,38]. In this context, consonant with our data, multiple lines of evidence indicate the ability of Iloprost to modulate oxidative markers at systemic levels [25,39,40,41]. Moreover, anti-inflammatory and immunomodulatory effects of Iloprost in SSc have been reported, which highlight its ability to reduce IL1, IL12, IL23, TNF, VEGF, and endothelin-1 and increase IL-2 and RANKL [42,43,44,45]. Interestingly, in line with our hypothesis, CTGF, a well-known profibrotic cytokine, which, in concert with TGF-β, plays a pivotal role in driving collagen overproduction [46,47], has been reported elevated in SSc patients and decreased after iloprost infusion [48]. The same results have also been shown for the synthesis of collagen in fibroblasts exposed to TGF-β [48].

Consistent with this, Gomez-Arroyo et al. also showed a reduced expression of CTGF in cardiac fibroblasts treated with iloprost [49], in addition to the increased activity of collagen synthesis mediators and ECM degradation such as metalloproteinase-9 (MMP9) [49]. Furthermore, a recent study showed an oxidative stress-induced increase in CTGF expression, which was attenuated by the use of antioxidants [50]. Finally, although not specifically in SSc, many other papers report the ability of Iloprost to modulate cytokines/chemokines production [51,52,53,54,55].

Along with the previously mentioned predisposing factors [3,4,5,6], the above-reported studies indicate oxidative stress as one of the important upstream signals responsible for driving SSc-fibrotic processes, making our hypothesis highly conceivable. Indeed, as previously reported [42,43,44,45], iloprost infusion in SSc patients can lower pro-inflammatory cytokines levels in SSc sera, which may drive the inhibition of type I collagen synthesis. It is also plausible that iloprost, concurrently with the decreasing of growth factors such as CTGF, TFG-β, and ET-1 [8,19,20,21], might also inhibit pro-oxidant factors released in the sera of SSc patients. Indeed, oxidative stress biomarkers, such as nitric oxide NO, malondialdehyde (MDA), and ROOH, resulted in elevated blood circulation in SSc patients compared to the healthy group [38]. On the contrary, the concentration of natural antioxidants, such as catalase (CAT) and superoxide dismutase SOD, instead appeared lower than in the control group [38,56]. In this regard, intravenous iloprost infusion has been shown to decrease the blood levels of pro-oxidant factors such as MDA [57] and improve the levels of natural antioxidants such as SOD and CAT [56]. Moreover, several other articles highlight the ability of Iloprost to modulate oxidative markers such as lipid peroxidation, glutathione, F8-Iso PGF2α [25,39,40,41]. In summary, several published papers have indicated the ability of Iloprost to modulate pro-fibrotic and oxidative markers at systemic levels. In this regard, our new data are consistent with the reported anti-inflammatory and immunomodulatory ability of Iloprost; therefore, we believe our proposed mechanicistic scenario highly conceivable.

## 3. Materials and Methods

### 3.1. Patients

Thirty-two serum samples were collected from two SSc patients’ groups (untreated and iloprost-treated) at the Unit of Complex Rheumatology, University of Sassari, Sassari, Italy. All SSc patients met the ACR/EULAR criteria for the classification of SSc [58]. The clinical and serological characteristics of the subjects enrolled in the study are summarized in Table 1. Iloprost-treated SSc patients underwent a monthly (5-day cycle—5–6 h/day) intravenous administration of the prostacyclin analog iloprost, at the greatest tolerated dose (0.5–2 ng/kg/min, mean dose 1.3 ng/kg/min); then, blood samples were taken after the 5-day cycle of iloprost infusion. Previous medications were maintained during the course of the study; no other drugs were started during the study period. Healthy donors were matched for gender, race, and smoking status. Study subjects were enrolled according to the protocol approved by the local ethics committee after signing the consent form. Healthy donors were recruited through posted flyers and enrolled after passing a screening questionnaire aimed at excluding the presence of any underlying vascular or autoimmune disease.

### 3.2. Cells Culture and Treatment

In this study, human pulmonary microvascular endothelial cells (HPMECs) isolated from the human lung of healthy donors were used (Innoprot, Bizkaia, Spain). Cells were cultured in HPMECs basal medium supplemented with endothelial cell growth supplement. When confluent, HPMECs were subcultured at a split ratio of 1:2 and used within three passages. Unless not specified in the text, cells were plated in 96-well black plates (Corning, Lowell, MA, USA) and processed for experiments in basal medium containing 10% (*v*/*v*) of the subjects’ sera. Sera among the different subjects were normalized based on protein content [17,59].

### 3.3. Intracellular ROS Determination

Intracellular ROS levels were assessed by using the ROS molecular probe 2′,7′-dichlorodihydrofluorescein diacetate (H_2_DCF-DA) (Molecular Probe, Eugene, OR, USA) as previously described with minor modification [59,60]. Within the cell, esterases cleave the acetate groups on H_2_DCF-DA, thus trapping the reduced form of the probe (H_2_DCF). Intracellular ROS oxidize H_2_DCF, yielding the fluorescent product DCF. For ROS measurements, cultured cells were preincubated for 30 min with PBS plus containing 10 μM H_2_DCFDA, then washed with PBS and treated as described in the figures’ legend. Fluorescence was measured using a Tecan GENios Plus microplate reader (Tecan, Mannedorf, Switzerland) in a light-protected condition. Excitation and emission wavelengths used for fluorescence quantification were 485 nm and 535 nm, respectively. Treatment-induced variation of fluorescence was kinetically measured over a time-course of 4 h. All fluorescence measurements were corrected for background fluorescence and protein concentration expressed as a means ± SD of the relative fluorescence unit (RFU) values.

### 3.4. Determination of Cell Viability

Cell viability was assessed in 96-well plates (BD Falcon) using the colorimetric 3-(4,5-dimethylthiazol-2-yl)-2,5-diphenyltetrazolium bromide (MTT reagent) assay (Promega, Madison, WI, USA) [61,62]. The yellow MTT reagent enters the cells and passes into the mitochondria, where mitochondrial dehydrogenases of viable cells cleave the tetrazolium ring, yielding reduced purple MTT formazan crystals, which are insoluble in aqueous solutions. This reduction occurs only when mitochondrial enzymes are active; therefore, conversion can be directly related to the number of viable cells. The formazan crystals can be dissolved in acidified isopropanol. The resulting purple solution is spectrophotometrically measured at 570 nm. After 24 h of treatment, 20 µL of MTT solution (2 mg/mL) in cell medium were added to the cells and incubated at 37 °C in a cell culture incubator for 2 h. At the end of the incubation period, the solution was removed, and the purple formazan product was solubilized with acidic isopropanol (0.04 N HCl in absolute isopropanol). Then, plates were analyzed at 570 nm using a GENios plus micro-plate reader (Tecan). Results are expressed as the mean ± SD of the absorbance units (ABS).

### 3.5. Determination of DNA Synthesis

DNA synthesis was assessed using a chemiluminescent immunoassay method, which is based on the measurement of BrdU incorporation during DNA synthesis (Cell Proliferation ELISA BrdU, Roche Applied Science). When cells are pulsed with BrdU, it is incorporated into newly synthesized DNA strands of actively proliferating cells. The incorporation of BrdU into cellular DNA can be detected using anti-BrdU antibodies, allowing an assessment of the population of cells synthesizing DNA. Subconfluent HPMECs were treated for 24 h, as indicated in the figure legends, and BrdU was added 12 h before the end of the experiments. After that, the culture supernatant was removed, and the cells were fixed with a Fix-Denat solution for 30 min. The Fix-Denat was discarded, and cells were incubated with an anti-BrdU antibody conjugated to horseradish peroxidase for 90 min. After rinsing three times with washing buffer, a peroxidase substrate solution was added and allowed to react for 3–10 min at room temperature. The horseradish peroxidase catalyzes the oxidation of diacyl hydrazide, where the reaction product decay from its excited state yields light. Finally, light emission was read using a GENios Plus microplate reader (Tecan). Results were normalized for protein content and expressed as the mean ± SD of the relative fluorescence units (RFU) values [62,63].

### 3.6. Production of Lentiviral Particles

A set of lentiviral particles was created to assess the synthesis of human collagen type-I (COL1A1) in transduced HPMECs exposed to the different experimental treatments. The lentivectors used to produce the lentiviral particles were pCOL1A1-tGFP, which is a plasmid containing a green fluorescent protein (GFP) under the control of human COL1A1 promoter, and pEFα-LV-FP602, which, instead, contains a red fluorescent protein (FP602) under the control of a constitutive promoter, the elongation factor 1-alpha (EFα) promoter. While activation of pCOL1A1-tGFP is associated with collagen synthesis, pEFα-LV-FP602 activation expresses a red fluorescent protein used as a control for normalizing the cell transduction efficiency. Lentiviral particles were prepared by transient transfection of the 293T/17 packaging cells, as previously described [64,65,66]. Briefly, when cell confluence was approximately 70%, a mix of transgene expression plasmid (pCOL1A1-tGFP or EFα-FP602) and the second generation packaging plasmids (pMD2.G and pCMVR8.74) were used to transfect the cells. The transfection was carried out using a calcium-phosphate solution consisting of a 1:1 mixture of 0.25 M of CaCl2:2X BBS (0.28 M NaCl, 0.05 M N,N-bis-(2-hydroxyethyl)-2-aminoethanesulfonic acid (BES), 1.5 mM Na2HPO4). The medium was exchanged 12 to 16 h post-transfection, and virus-containing media was harvested at 24 h intervals twice, beginning 24 h after changing the medium. The collected medium was spun in a SW28 rotor for 2 h at 19,400 rpm in a L8-80M ultracentrifuge (Beckman); then, the pellet was re-suspended in 1 mL HBSS and spun again in the SW55 rotor for 2 h at 21,000 rpm in a L8-80M ultracentrifuge (Beckman). Finally, the second pellet was re-suspended in 200 μL in HBSS, and the virus suspension was vortexed for 1 to 2 h at low speed before storing at −80 °C in 20 μL aliquots. Virus titer was determined by the p24 ELISA kit (PerkinElmer) according to the manufacturer.

### 3.7. Generation of HPMEC/pCol1GFP-pEFα-FP602 Stable Cell Lines

Generation of stable cell line containing both pCOL1A1-tGFP and EFα-FP602 was carried out as previously described [61,66,67]. Briefly, 50–60% confluent HPMECs were transduced with the produced lentiviral particles using the optimized multiplicity of infection to reach an infection efficiency of nearly 95%. Cells were then incubated for 24 h; then, an equal amount of fresh medium with no virus was added for an additional 24 h. At around 72–96 h, the efficiency was measured by flow cytometer analysis. Stable transduced HPMECs were used for assessing the COLIA synthesis under the different experimental conditions.

### 3.8. Collagen Determination

As previously described, COLIA synthesis was investigated, employing the produced HPMEC/pCol1GFP-pEFα-FP602 stable cell lines [17,59]. This method allows us to perform the real-time assessment of multiple samples at the same time in a 96-well plate using a small amount of subject sera [17,59]. HPMEC/pCol1GFP-pEFα-FP602 cells were exposed to basal medium containing 10% (*v*/*v*) of sera from SSc patients (SSc), 10% sera from iloprost-treated SSc patients (SSc + I), and 10% sera from healthy donors (HD). Treatment-induced variation of GFP fluorescence was kinetically measured over a time-course of 4 h using a Tecan GENios Plus microplate reader (Tecan, Mannedorf, Switzerland). Excitation wavelengths used for fluorescence quantification were 485 nm and 535 nm, while emission wavelengths were 535 nm and 590 nm for pCOL1A1-LV-tGFP and EF*α*-LV-FP602, respectively. Data were normalized for transduction efficiency by reporting the ratio of pCOL1A1-LV-tGFP to EF*α*-LV-FP602 and expressed as a means ± SD of the relative fluorescence unit (RFU) values.

### 3.9. Statistical Analysis

Kruskall–Wallis one-way analysis of variance, followed by a post-hoc Dunn’s test for multiple comparisons, was used to detect differences among the studied groups. All statistical analyses were performed using GraphPad Prism version 9.00 for Windows (GraphPad Software, San Diego, CA, USA), and *p*-values < 0.05 were considered to be statistically significant.

## 4. Conclusions

This study supports the hypothesis that pro-oxidant circulating factors activate collagen synthesis and drive endothelial dysfunction initiation and progression, ultimately leading to SSc pathological vascular alterations. In addition, our results demonstrate that prostacyclin (or prostacyclin analogs) employment improves ECs oxidative status and prevents collagen synthesis and ECs injury, thus providing new clues concerning the mechanisms of action of iloprost and its clinical benefits in terms of vascular damage.

## Figures and Tables

**Table 1:** Patient demographics and clinical characteristics.

Variables	SSc (*n* = 32)	HD (*n* = 14)
Age at serum sampling (years) *	55 ± 11	54.1 ± 10.4
Sex		
Female	24 (75)	12 (86)
Male	8 (25)	2 (14)
Race		
White	30 (94)	11 (79)
Black	2 (6)	3 (21)
Smoking status		
Never	14 (44)	10 (71)
Current	10 (31)	4 (29)
Past	8 (25)	0
SSc types		
Diffuse	22 (69)	
Limited	10 (31)	
SSc duration (years) *	13 ± 12.3	
Autoantibody status		
Anti RNA polymerase 3	4 (6)	
Anti Scl-70	20 (63)	
Anti ACA	4 (13)	
Medication use (current)		
Immunosuppressants ‡	4 (13)	
Calcium channel blocker	16 (50)	
Endothelin receptor antagonist	4 (13)	
Phosphodiesterase 5 inhibitor	2 (13)	
Prostanoid	20 (63)	
Statin	2 (6)	
Aspirin	8 (25)	

All values are given as number (%) unless otherwise specified. * Mean ± SD. SSc, systemic sclerosis; HD, healthy donors; ACA, anticentromere antibody; Scl-70, topoisomerase I; ‡ Use of immunosuppressants include cyclophosphamide, mycophenolate, methotrexate, hydroxycholorquine, or prednisone.

## Data Availability

The data presented in this study are available in this article.

## Sample Availability

Samples of the compounds are not available from the authors.
